# Allosteric Modulation of the Calcium-Sensing Receptor

**DOI:** 10.2174/157015907781695982

**Published:** 2007-09

**Authors:** Anders A Jensen, Hans Bräuner-Osborne

**Affiliations:** Department of Medicinal Chemistry, Faculty of Pharmaceutical Sciences, University of Copenhagen, Universitetsparken 2, 2100 Copenhagen, Denmark

**Keywords:** Calcium-sensing receptor, CaR, allosteric modulator, calcimimetic, calcilytic, cinacalcet, hyperparathyroidism.

## Abstract

The calcium (Ca^2+^)-sensing receptor (CaR) belongs to family C of the G-protein coupled receptors (GPCRs). The receptor is activated by physiological levels of Ca^2+^ (and Mg^2+^) and positively modulated by a range of proteinogenic L-α-amino acids. Recently, several synthetic allosteric modulators of the receptor have been developed, which either act as positive modulators (termed calcimimetics) or negative modulators (termed calcilytics). These ligands do not activate the wild-type receptor directly, but rather shift the concentration-response curves of Ca^2+^ to the left or right, respectively. Like other family C GPCRs, the CaR contains a large amino-terminal domain and a 7-transmembrane domain. Whereas the endogenous ligands for the receptor, Ca^2+^, Mg^2+^ and the L-α-amino acids, bind to the amino-terminal domain, most if not all of the synthetic modulators published so far bind to the 7-transmembrane domain.

The most prominent physiological function of the CaR is to maintain the extracellular Ca^2+^ level in a very tight range *via* control of secretion of parathyroid hormone (PTH). Influence on e.g. secretion of calcitonin from thyroid C-cells and direct action on the tubule of the kidney also contribute to the control of the extracellular Ca^2+^ level. This control over PTH and Ca^2+^ levels is partially lost in patients suffering from primary and secondary hyperparathyroidism. The perspectives in CaR as a therapeutic target have been underlined by the recent approval of the calcimimetic cinacalcet for the treatment of certain forms of primary and secondary hyperparathyroidism. Cinacalcet is the first clinically administered allosteric modulator acting on a GPCR, and thus the compound constitutes an important proof-of-concept for future development of allosteric modulators on other GPCR drug targets.

## INTRODUCTION

Calcium (Ca^2+^) is an essential intracellular messenger and a mediator of a wide range of important physiological and pathophysiological cellular processes. The intracellular concentration of Ca^2+^ ([Ca^2+^]_i_) is at a tightly controlled equilibrium between the actions of various voltage- and ligand-gated calcium ion channels and the release of Ca^2+^ from intracellular stores, for example as a result of the signalling of certain G-protein coupled receptors (GPCRs). Within the last two decades it has been realized that several cell types also are able to ‘sense’ fluctuations in the extracellular Ca^2+^ concentration ([Ca^2+^]_o_) and that even minute changes in [Ca^2+^]_o_ elicits an array of cellular responses, such as an increase in [Ca^2+^]_i_ and the release of various hormones [7, 10, 34, 76]. The cloning of the calcium-sensing receptor (CaR), a GPCR mediating the intracellular responses to extracellular stimuli in the form of Ca^2+^ (and Mg^2+^), in 1993 unequivocally established Ca^2+^ as a first messenger as well as a second messenger [9]. In the subsequent years CaR has been shown to be a unique regulator of metabolic processes in bone, kidney, parathyroid and many other tissues [7, 10, 34, 76]. Thus, the receptor is an attractive target for the treatment of several disorders, and the therapeutic prospects in CaR ligands have been underlined by the recent introduction of cinacalcet (Sensipar^®^ and Mimpara^®^ in USA and Europe, respectively) in the clinical treatment of uremic secondary hypercalcemia parathyroidism and parathyroid cancer [10, 76]. In recent years the perception of CaR as a metabolic sensor has been substantiated and further expanded by the realization that CaR signalling is potentiated by several L-α-amino acids and by the recent cloning of a related GPCR, GPRC6A, which also mediates the signalling of L-α-amino acids and divalent cations [13, 17, 45, 61, 78].

The physiological importance of Ca^2+^ sensing and the role of CaR in health and disease have been outlined in several excellent reviews, to which the reader is referred [10, 34, 76]. Following a brief introduction to the structure and signal transduction of CaR, the present review will primarily focus on the pharmacology of endogenous and synthetic allosteric modulators of CaR and their binding modes at the receptor. Finally, the physiological roles played by CaR, and the therapeutic prospects for cinacalcet, the first and only allosteric modulator of a GPCR to ever enter the drug market will be discussed. 

## MOLECULAR STRUCTURE AND SIGNAL TRANSDUCTION OF THE CAR

**Fig. (1):**
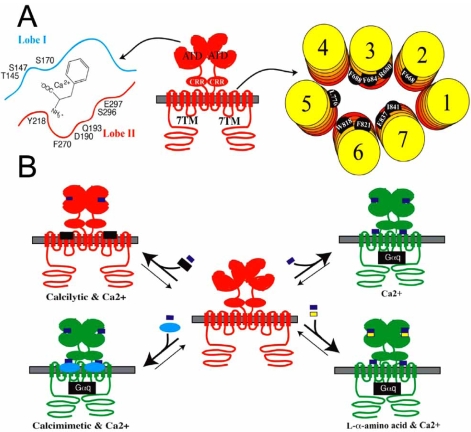
**A**. The homodimeric CaR complex. The ATD, CRR and 7TM regions of the receptor are indicated. The residues in the ATD involved in Ca^2+^ and L-α-amino acid binding and the residues in the 7TM involved in the binding of calcimimetics and calcilytics are given. **B**. The signal transduction through the CaR homodimer when exposed to Ca^2+^ alone, Ca^2+^ and L-α-amino acids, Ca^2+^ and calcilytics, and Ca^2+^ and calcimimetics. Inactive and active conformations of the CaR homodimer is given in red and green, respectively.

The CaR belongs to family C of the GPCR superfamily, a small subfamily comprised by receptors mediating the intracellular metabolic responses to extracellular stimuli in the form of nutrients such as amino acids, ions and taste molecules. Besides CaR family C is constituted by eight metabotropic glutamate receptors (mGluRs), two γ-aminobutyric acid_B_ receptors (GABA_B_Rs), several taste receptors and the recently cloned promiscuous L-α-amino acid receptor GPRC6A [3, 10, 19, 34, 37, 45, 51, 52, 76, 78, 84]. The family C GPCR does not exhibit significant amino acid sequence homology with the other GPCRs, and it shares very few fingerprint residues with other receptors in the superfamily. However, the family C GPCR has the same overall topology as all other GPCRs, as it is constituted by an extracellular amino-terminal domain (ATD), seven transmembrane α-helices (TM1-TM7) connected by intra- and extracellular loops (this region will be referred to as ‘the 7TM’), and an intracellular carboxy-terminal (Fig. **1A**). Finally, a small region containing nine highly conserved cysteine residues, termed the cysteine-rich region (CRR), is connecting the ATD and the 7TM of CaR.

The family C GPCRs exist as constitutive dimeric (or oligomeric) receptor complexes in the cell membrane (Fig. **1A**). Whereas the GABA_B_ and taste receptors exist as heterodimeric receptors composed of two different subunits, CaR and the mGluRs form homodimeric complexes *via* several covalent and noncovalent interactions between the two subunits [2, 3, 47, 52, 57, 67, 72, 73, 77, 84]. The most remarkable structural feature of the family C GPCR is its extraordinarily large extracellular ATD, which consists of ~600 amino acid residues and contains the orthosteric site of the receptor, i.e. the binding site of the endogenous agonist [6, 31]. The crystal structure of the ATD of the mGluR subtype 1 has brought considerable insight into the structure of this region [40, 47, 77]. The ATD consists of two globular lobes arranged in a clam shelf structure, and thus the domain is often referred to as a ‘Venus Flytrap Domain’. The orthosteric site is situated in the cleft between these two lobes, where the agonist binds *via* interaction to residues located on both sides of this cleft. 

The location of the orthosteric site in the family C GPCR contrasts that of the rhodopsin-like family A GPCR, where the endogenous agonist binds to a pocket situated within the 7TM of the receptor. Furthermore, G-protein coupling to the family C receptor also occurs to different intracellular receptor regions than to the family A GPCR [62]. Thus, the molecular events underlying signal transduction through the family C GPCR appear to be quite different from those involved in the signalling of other GPCRs, and the fact that the receptors are dimeric complexes seems to be of key importance for their signal transduction [62]. Agonist binding to each of the clefts in the two ATDs of the homodimeric family C GPCR causes the two regions to close up, which in turn elicits a conformational twist in the entire receptor complex believed to rearrange the composition of two 7TM regions and hereby enabling G-protein coupling to the receptor complex (Fig. **1B**) [38, 62].

### The Orthosteric Site(s) in CaR

In the crystal structure of the mGluR1 ATD, L-glutamate binds to the cleft formed by the two lobes through interactions with 13 residues distributed on both sides of the cleft [47, 77]. The endogenous agonists for CaR, Ca^2+^ and Mg^2+^, have also been demonstrated to bind to the ATD of the receptor [6, 31], and mutations of several of the residues in CaR corresponding to mGluR1 residues involved in agonist binding have been shown to impact Ca^2+^- and Mg^2+^-induced signalling through the receptor dramatically [6, 83]. Based on these mutagenesis studies and a homology model of the CaR ATD based on the mGluR1 ATD crystal structure, Ruat and colleagues have recently proposed that Ca^2+^ binds to CaR by coordination to the polar residues Ser^170^, Asp^190^, Gln^193^, Ser^296^ and Glu^297^ with minor contributions from residues Ser^147^, Tyr^218^ and Phe^270^ (Fig. **1A**) [74]. 

The signal transduction through CaR is characterized by a remarkable high cooperativity with Hill coefficients of 3-4 and 2-3 for Ca^2+^ and Mg^2+^, respectively [6, 31]. This and the smaller sizes of Ca^2+^ and Mg^2+^ ions compared to glutamate, GABA and other agonists for family C GPCRs originally prompted speculations that the orthosteric site in the ATD has to be occupied by more than one Ca^2+^ or Mg^2+^ ion in order for CaR to become activated. However, subsequently a small segment in the carboxy terminal of CaR has been proposed to control receptor densitization and influence the cooperativity [24]. Furthermore, in addition to its binding site in the ATD Ca^2+^ has recently been proposed to act as an agonist at a site situated in the 7TM of the receptor [68]. The presence of orthosteric sites in both the ATD and the 7TM of the receptor would certainly explain its high cooperativity. 

## ALLOSTERIC MODULATION OF CaR

### Endogenous Allosteric Modulators of CaR

*L-α-amino acids.* The CaR appears to be highly susceptible to allosteric modulation by a wide range of endogenous ligands and environmental conditions. For example, Na^+^ ion concentration and ion strength [65] as well as changes in environmental pH [64] have been shown to influence CaR signalling significantly. More importantly, however, CaR signalling has recently been shown to be potentiated by numerous L-α-amino acids, in particular the aromatic amino acids L-phenylalanine, L-tyrosine, L-histidine and L-tryptophan [17]. The L-α-amino acids potentiate CaR signalling at physiologically relevant concentrations, and they have been shown to inhibit PTH secretion from human parathyroid cells in an acute and reversible manner [16]. The dual activity of amino acids and Ca^2+^ at CaR appears to be a feature shared by many family C GPCRs [15]. Several mGluRs and the GABA_B_ receptors have been shown to possess Ca^2+ ^sensing properties [46, 63, 80], and the signalling of the promiscuous L-α-amino acid receptor GPRC6A is also potentiated by Ca^2+^ and Mg^2+^ [13, 61]. Interestingly, the L-α-amino acids have been demonstrated to exert their effects on CaR signalling through binding to an allosteric site situated in the close vicinity of the orthosteric site in the ATD of the receptor, in fact some receptor residues are involved in both Ca^2+^ and the L-α-amino acid binding (Fig. **1A**) [49, 50, 83]. A triple alanine mutation of the adjacent serine residues Ser^169^, Ser^170^ and Ser^171^, of which Ser^170^ has been shown to be crucial for Ca^2+^ signalling through CaR, eliminates the potentiation of receptor signalling exerted by L-α-amino acids [83], whereas introduction of the double mutation T145A/S170T in CaR eliminates the ‘L-α-amino acid sensing’ ability of CaR while Ca^2+^ displays unaltered potency at the mutant compared to the wild type receptor [49]. The apparent concomitant binding of Ca^2+^ and the L-α-amino acid to two distinct sites both situated in the cleft between the two lobes of the ATD in CaR is in good agreement with studies of mGlu and GABA_B_ receptors, where Ca^2+^ binding has been shown to take place to neighbouring residues to those involved in L-glutamate and GABA binding, respectively [46, 63]. Furthermore, the realization that CaR is a receptor for both amino acids and divalent cations may also explain why the binding cavity in the ATD of CaR, according to molecular modelling, is of a similar size as those in the mGlu and GABA_B_ receptors [15].

The L-α-amino acids were originally reported to be true allosteric potentiators of CaR in the sense that they were found to be unable to activate CaR in the absence of Ca^2+^ or other CaR agonists [17]. Subsequently, however, another group has claimed that L–phenylalanine is a CaR agonist capable of eliciting signalling in the absence of calcium [71]. Interestingly, Ca^2+^ and the L-α-amino acids appear to elicit different intracellular Ca^2+^ oscillations through CaR, indicating that the receptor response may differentiate depending on the nature of the external stimuli [71, 82]. Ca^2+^-mediated CaR signalling gives rise to high-frequency sinusoidal oscillations upon a raised plateau of [Ca^2+^]_I_ through a phospholipase C/inositol trisphosphate pathway, and the frequency of these oscillations are negatively regulated by phosphorylation of the intracellular Thr^888^ residue in the carboxy terminal of CaR by protein kinase C [71, 82]. In contrast, stimulation of the receptor with L-α-amino acids, on the other hand, induces transient oscillations characterized by repetitive, low frequency [Ca^2+^]_i_ spikes that return to the baseline level [71, 82]. These spikes are not induced *via* phospholipase C/inositol trisphosphate pathway but *via* the transient receptor potential channel TRPC1 *via* a signalling cascade involving Gα_12_ proteins, the GTPase Rho, filamin-A and the actin cytoskeleton [70, 71]. In order to explain the different intracellular responses caused by Ca^2+^ and L-α-amino acids, the two types of agonists have been proposed to stabilize different active conformations of CaR [71]. Considering the close proximity of the binding sites for Ca^2+^ and the L-α-amino acids in the ATD and the considerable distance from these binding sites to the intracellular G-protein coupling regions of CaR, it is quite remarkable that the two agonists elicit distinct signalling cascades.

### Synthetic Allosteric Modulators of the CaR

Besides its endogenous agonists Ca^2+^ and Mg^2+^, CaR is also activated by other inorganic ions like Gd^3+^ and Ba^2+^, polyamines like spermine and spermidine and by the antibiotic neomycin [6, 31, 66]. Explorations into the therapeutic prospects of CaR have been complicated by the chemical nature of these agonists, since they are not obvious lead candidates for medicinal chemistry development. However, this obstacle has been circumvented by the development of several organic compounds modulating CaR signalling *via* binding to allosteric sites in the receptor. Based on their pharmacological properties on CaR signalling, these synthesised allosteric modulators are divided into two classes: calcimimetics and calcilytics. Calcimimetics are allosteric potentiators (or positive allosteric modulators) of CaR, the name referring to their ability to mimic the actions of calcium, whereas calcilytics are allosteric inhibitors (or negative allosteric modulators) of the receptor. 

#### Calcimimetics

**Fig. (2):**
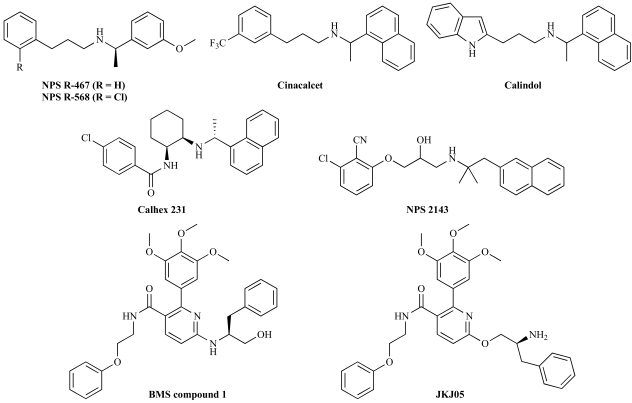
Chemical structures of synthetic allosteric modulators of CaR.

The phenylalkylamine structure fendiline is a relatively weak allosteric potentiator of CaR, which has been used as the lead for the development of a series of more potent calcimimetics, including the compounds NPS R-467 and NPS R-568 (Fig. **2**) [56]. NPS R-568 has been shown to decrease PTH secretion and increase calcitonin secretion through its activity at CaR on parathyroid cells and C cells, and these effects leads to hypocalcemia due to a reduced efflux of Ca^2+^ from bone [22, 23, 56]. NPS R-568 and NPS R-467 have been further developed into cinacalcet, which recently has entered the clinic for the treatment of uremic secondary hypercalcemia parathyroidism and parathyroid cancer (see below) [55]. In another series of calcimimetics, medicinal chemistry optimization of N-arylsulfon-1,2-diamine and N-aryl-1,2-diamine structures have resulted in the development of calindol [(*R*)-2)-[*N*-(1-(1-naphtyl)ethyl)aminomethyl]indole] [20, 42]. As can be seen from (Fig. **2**), the structures of the NPS calcimimetics, cinacalcet and calindol are very similar.

#### Calcilytics

In spite of some structural similarity to NPS R-568, the calcilytic NPS 2143 (*N*-[(R)-2-hydroxy-3-(2-cyano-3-chlorophenoxy) propyl]-1,1-dimethyl-2-(2-naphtyl)ethylamine) was developed based on a hit identified in a high throughput screening (Fig. **2**) [54]. Being the first published CaR inhibitor, the compound has become an important pharmacological tool to study the physiological functions governed by the receptors [54]. In agreement with the observed effects of calcimimetics, NPS 2143 has been shown to increase PTH secretion from parathyroid cells and to increase plasma levels of Ca^2+^. A considerable amount of structure-activity work has been performed using NPS 2143 as template. However, variations in the 1,1-dimethyl-2-naphtalen-2-yl-ethylamine part of the molecule have not resulted in more potent calcilytics [26, 81]. Another calcilytic is Calhex 231 (*N^1^*-(4-Chlorobenzoyl)-*N^2^*-[1-(1-naphtyl)ethyl]-*trans*-1,2-diaminocyclohexane), one of the most potent CaR inhibitors published to date [41]. Finally, a series of structurally distinct calcilytic compounds not based on the phenylalkylamine-structure have recently been published [1, 35].

### Allosteric Sites in the 7TM of CaR

The 7TM of the family C GPCR seems to be an attractive target for allosteric modulators, since a plethora of allosteric modulators of mGlu and GABA_B_ receptors have been demonstrated to target this region [21,25, 39]. Analogously, many of the published synthetic allosteric modulators of the CaR have been shown to target the 7TM of the receptor [30, 32, 36, 48, 59, 60], and the residues involved in the binding of the calcilytics NPS 2143 and Calhex 231 and the calcimimetics NPS R-568 and calindol have been outlined in further detail in mutagenesis studies based on homology models of the 7TM of CaR built using the crystal structure of the family A GPCR rhodopsin as template (Fig. **1A**) [48, 59, 60]. NPS 2143 and Calhex 231 both bind to the extracellular part of the 7TM of CaR, and their binding sites are largely overlapping although minor differences in the binding modes of the two calcilytics exist. The binding of both ligands is anchored in a salt bridge from the protonated secondary amino group in the ligands to the Glu^837^ residue in TM7 [36, 48, 60]. In addition to this interaction, the aromatic ring systems in NPS 2143 is believed to form hydrophobic contacts and π -stacking with the TM2 residue Phe^668^ and the TM3 residues Phe^684^ and Phe^688^, and the Arg^680^ residue in TM3 has been proposed to interact with the hydroxy group of the ligand [48, 59]. Finally, mutation of Ile^841^ located one α-helix turn below Glu^837^ in TM7 of CaR has also been found to result in reduced inhibitory potency of NPS 2143 [59]. Phe^684^, Phe^688^ and Ile^841^ are also important for Calhex 231 binding to CaR but the inhibitory effects of this calcilytic is not affected by an alanine mutation of Arg^680^, and furthermore it is less affected by a F688A mutation in CaR than NPS 2143. On the other hand, the antagonistic potency of Calhex 231 is reduced by an alanine mutation of Trp^818^ in TM6 of CaR, whereas that of NPS 2143 is not [59]. Finally, alanine substitutions of Leu^776^ and Phe^821^ in TM5 and TM6 of CaR, respectively, result in increased antagonistic potency of Calhex 231, whereas NPS 2143 does not display changes in its IC_50_ values at these mutants compared to the wild type receptor [59].

The binding of the two calcimimetics NPS R-568 and calindol to CaR are also centered around an ionic interaction between the charged nitrogens of the compounds and Glu^837^ in TM7 [59]. However, in contrast to their involvement in calcilytic binding, the TM3 residues Arg^680^, Phe^684^ and Phe^688^ are not important for the binding of the calcimimetics. Instead, TM6 has been proposed of key importance for their ability to potentiate CaR signalling, as the potentiating effects on CaR signalling by NPS R-568 and calindol are eliminated by mutations of the Trp^818^ residue and the Phe^821^ residue, respectively [59].

Considering that NPS R-568, NPS 2143, Calindol and Calhex 231 are structurally related phenylalkylamines all having a positively charged amino group, it is not surprising that they target a common allosteric site in the 7TM of CaR. Interestingly, a structurally distinct calcilytic from Bristol-Meyers Squibb (‘BMS compound 1’ in Fig. **2**) has recently been shown not to compete with the binding of a tritiated NPS 2143 analogue to CaR, indicating that this compound has a different site of action than NPS 2143 and the other phenylalkylamines [1]. In support of this, the Spiegel group has reported that binding of the compound to CaR does not involve the Glu^837^ residue in TM7 shown to be crucial for the binding of all of the phenylalkylamine-based allosteric modulators, whereas mutation of the Ile^841^ residue eliminates the inhibitory effects of BMS compound 1 on CaR signalling [35]. Hence, this calcilytic appear to bind to an allosteric site in the 7TM of CaR, which is in close proximity to but does not overlap with the site for the phenylalkylamine-based modulators. Furthermore, the fact that the allosteric inhibitor JKJ05, a closely related analogue of BMS compound 1, was converted into an allosteric potentiator with the introduction of the E837A mutation into CaR, further illustrates the subtle molecular differences in the 7TM conformations stabilized by calcilytics and calcimimetics [35].

It is interesting that the four transmembrane helices involved in agonist binding to the family A GPCR, TM3, TM5, TM6 and TM7, also form the binding pocket for the allosteric modulators in CaR and other family C GPCRs. This raises the question whether the allosteric potentiators in fact are allosteric agonists with intrinsic activities at CaR in the absence of Ca^2+^ and Mg^2+^, as it has been reported to be the case for allosteric potentiators of mGlu and GABA_B_ receptors [ 4, 28]. In a recent study, calindol has been demonstrated to be an agonist at a CaR construct, where the ATD and the carboxy terminal of the receptor have been truncated [69]. Although no calcimimetics have displayed intrinsic activity at the wild type receptor the agonism displayed by calindol at this artificial CaR construct underlines the similarities between the orthosteric site in the rhodopsin-like family A GPCR and the common allosteric site for the phenylalkylamine-based calcimimetics and calcilytics in CaR. Whereas all published allosteric modulators of CaR appear to bind to the 7TM of the receptor, synthetic allosteric modulators targeted to the ATD of the receptor could also be envisioned. As mentioned below, a considerable number of somatic mutations in the CaR gene linked to various disorders have been shown to alter the signalling properties of the receptor, and this indicates that the entire receptor protein is susceptible to allosteric modulation. Furthermore, the allosteric potentiation of CaR signalling by L-α-amino acid binding to a site close to the orthosteric site in the ATD further supports this notion. 

## PHYSIOLOGICAL ROLES OF CAR

Due to the significance of calcium as a primary and secondary messenger, the extracellular calcium level in the blood is very tightly controlled *via* regulation of dietary uptake, renal excretion and bone metabolism. Thus an elevated calcium level will lead to decreased renal calcium resorption, decreased intestinal absorption, increased bone formation and decreased bone resorption, which at least to some extend is caused either directly or indirectly (*via* parathyroid hormone (PTH) and calcitonin hormones) by activation of CaR [8]. In accordance with the broad expression of CaR, the receptor might also possess other important physiological roles such as regulation of gut hormone secretion [14] and control of arterial blood pressure [79], but these roles remains to be studied in details.

In the parathyroid glands, CaR has been identified as a central regulator of the synthesis and secretion of PTH and of parathyroid cellular proliferation. Thus, a decrease in serum calcium level will lead to increased secretion of PTH, which in turn will act on (1) the kidney to promote calcium reabsorption and synthesis of 1,25(OH)_2_D_3_ (a derivative of vitamin D_3_ which promotes intestinal absorption of calcium and phosphate) and (2) bone to release skeletal calcium. These effects are further augmented by concomitant decrease in secretion of calcitonin from thyroid C-cells, which has the opposite effect of PTH on bone metabolism and kidney reabsorption [8]. CaR is also expressed in the kidney, bone and intestine and might thus also contribute directly to the physiological effects outlined above in addition to the indirect effects *via* PTH and calcitonin, but the significance of these direct effects are still being debated intensely.

The physiological importance of CaR on calcium homeostasis and PTH secretion has been elucidated by analysis of genetically modified mice and analysis of humans with inherited or acquired disorders involving the CaR. More recently, the use of calcimimetics and calcilytics *in vivo* has also contributed significantly to our knowledge (see next section).

CaR knock-out mice display highly elevated PTH levels, despite significantly elevated serum calcium levels, and parathyroid hyperplasia [33], which all point to the direct effects of CaR on the control of PTH secretion and parathyroid cellular proliferation. A second line of evidence comes from identification of numerous mutations in the CaR gene identified from individuals suffering from three different genetic disorders, Familial Hypocalciuric Hypercalcemia (FHH), Neonatal Severe Hyperparathyroidism (NSHPT) and Autosomal Dominant Hypocalcemia (ADH). Pharmacological characterization of these somatic mutations in CaR mutants heterogeneously expressed in mammalian cell lines have shown that introduction of the FHH and NSHPT mutations in CaR result in reduced potencies of Ca^2+^ at the receptor, whereas ADH mutants display increased Ca^2+^ potencies. Collectively, the studies provide a compelling explanation for the clinically observed elevated and decreased serum calcium levels in FHH/NSHPT and ADH, respectively [12]. A third line of *in vivo* evidence comes from autoimmunity disorders in which patients have developed anti-CaR antibodies that either inhibit [44] or activate [43] CaR and thereby produce clinical manifestations resembling FHH or ADH, respectively. Finally, patients with either primary or uremic secondary hyperparathyroidism (PHPT and SHPT, respectively) show significant reduced expression of CaR in the parathyroid gland [18, 27], which at least in part provide a possible explanation for the observed increase in PTH secretion. 

## THERAPEUTIC PROSPECTS

Recently, North American and European drug regulatory authorities approved the calcimimetic cinacalcet for the treatment of SHPT in patients with end-stage renal disease receiving hemodialysis and of PHPT caused by parathyroid cancer. Both diseases are characterized by elevated PTH and calcium levels as well as other disturbances in mineral metabolism, which leads to bone diseases and other complications [11, 75]. Several clinical trials have been conducted with cinacalcet and other calcimimetics, which have shown that the compounds are very effective in reducing the elevated PTH and calcium levels in various forms of PHPT and SHPT [5, 8, 58]. So far the drug has only been approved for the previously mentioned forms of PHPT and SHPT, but it is predicted that the drug will also be approved for other forms of hyperparathyroidism in the future [8]. In addition the drug might also be approved for the treatment of FHH, NSPHP and hypercalcemia caused by autoantibodies that inhibit the CaR [8]. 

Only few pre-clinical studies have been conducted with calcilytics and none have yet been approved for human use. Two studies with NPS 2143 have shown that the compound leads to a 3-4 fold increase in PTH levels lasting for several hours causing an increase in bone turnover, but not an increase in bone mineral density (BMD) [29, 53]. Given that injections of PTH is approved for the treatment of osteoporosis, it has been suggested that short acting calcilytics, causing bursts of PTH levels similar to the injected PTH rather than the sustained increases seen with NPS 2143, could lead to increases in BMD [29, 53]. BMS compound 1 has such a short acting profile *in vivo* in rats resulting in PTH bursts [1], but the effect of the compound on BMD has yet to be studied and the hypothesis of use of calcilytics for treatment of osteoporosis thus remains to be proven. Other putative indications for calcilytics are treatment of ADH and hypocalcemia caused by autoantibodies that activate the CaR [8]. 

## CONCLUSIONS

Since the cloning of the CaR in 1993 a wealth of information on its structure, function, pharmacology and physiology has been generated. The receptor has been shown to be a central regulator of the secretion of PTH and an important feed-back sensor to control serum calcium levels, and this has formed the rationale for the development of cinacalcet for treatment of certain forms of PHPT and SHPT. Cinacalcet is a positive allosteric modulator of CaR and is the first compound with such a pharmacological profile on a GPCR to obtain regulatory approval as a drug. As such, the compound is very important as it has demonstrated proof-of-concept for the many other allosteric GPCR modulators that are currently in drug development. It is to be expected that the indications of cinacalcet will be broadened in the future, and that other calcimimetics will be marketed. Whether a calcilytic will also reach the market to treat e.g. osteoporosis is less certain as additional pre-clinical studies using short-acting analogues are needed to show proof-of-concept. 

So far, the physiological function of CaR is best known in the parathyroid gland and the kidney. However, the receptor shows a much wider expression pattern and additional physiological functions have yet to be fully investigated. The development of allosteric modulators that can be applied *in vitro* and *in vivo* will undoubtedly accelerate such studies as exemplified by a recent study suggesting a role of CaR in the control of arterial blood pressure [79]. In addition the physiological importance of the allosteric modulation of CaR by L-α-amino acids remains to be fully elucidated. In that respect, it would be interesting to further elucidate this allosteric site by characterization of series of analogues of the aromatic L-α-amino acids, which could hopefully lead to development of potent and selective compounds that could be used to study the physiological importance of L-α-amino acid sensing by the CaR. Furthermore, considering the reports demonstrating differential CaR signaling exerted by Ca^2+^ and L-α-amino acids [68, 69, 80], different calcimimetics could be envisioned to elicit different physiological responses. Finally, albeit higher levels of Ca^2+^ or Mg^2+^ are needed to modulate/activate GPRC6A than CaR [13, 45, 61] it is possible that GPCR6A has a physiological role as a divalent cation sensor in e.g. bone where higher concentrations are present than in blood. Further studies using selective ligands and/or genetically modified mice are needed to elucidate the physiological role of this novel receptor.

## References

[1] Arey BJ, Seethala R, Ma Z, Fura A, Morin J, Swartz J, Vyas V, Yang W, Dickson JK Jr, Feyen JH (2005). A novel calcium-sensing receptor antagonist transiently stimulates parathyroid hormone secretion *in vivo*. Endocrinology.

[2] Bai M, Trivedi S, Brown EM (1998). Dimerization of the extracellular calcium-sensing receptor (CaR) on the cell surface of CaR-transfected HEK293. J. Biol. Chem.

[3] Bettler B, Kaupmann K, Mosbacher J, Gassmann M (2004). Molecular structure and physiological functions of GABA_B_ receptors. Physiol. Rev.

[4] Binet V, Brajon C, Le Corre L, Acher F, Pin JP, Prezeau L (2004). The heptahelical domain of GABA_B2_ is activated directly by CGP7930 a positive allosteric modulator of the GABA_B_ receptor. J. Biol. Chem.

[5] Block GA, Martin KJ, de Francisco AL, Turner SA, Avram MM, Suranyi MG, Hercz G, Cunningham J, Abu-Alfa AK, Messa P, Coyne DW, Locatelli F, Cohen RM, Evenepoel P, Moe SM, Fournier A, Braun J, McCary LC, Zani VJ, Olson KA, Drueke TB, Goodman WG (2004). Cinacalcet for secondary hyperparathyroidism in patients receiving hemodialysis. N. Engl. J. Med.

[6] Bräuner-Osborne H, Jensen AA, Sheppard PO, O'Hara P, Krogsgaard-Larsen P (1999). The agonist-binding domain of the calcium-sensing receptor is located at the amino-terminal domain. J. Biol. Chem.

[7] Brown EM (1991). Extracellular Ca^2+^ sensing, regulation of parathyroid cell function, and role of Ca^2+^ and other ions as extracellular (first) messengers. Physiol. Rev.

[8] Brown EM (2007). Clinical lessons from the calcium-sensing receptor. Nat. Clin. Pract. Endocrinol. Metab.

[9] Brown EM, Gamba G, Riccardi D, Lombardi M, Butters R, Kifor O, Sun A, Hediger MA, Lytton J, Hebert SC (1993). Cloning and characterization of an extracellular Ca^2+^-sensing receptor from bovine parathyroid. Nature.

[10] Brown EM, MacLeod RJ (2001). Extracellular calcium sensing and extracellular calcium signaling. Physiol. Rev.

[11] Chan AK, Duh QY, Katz MH, Siperstein AE, Clark OH (1995). Clinical manifestations of primary hyperparathyroidism before and after parathyroidectomy. A case-control study. Ann. Surg.

[12] Chattopadhyay N, Brown EM (2006). Role of calcium-sensing receptor in mineral ion metabolism and inherited disorders of calcium-sensing. Mol. Genet. Metab.

[13] Christiansen B, Hansen KB, Wellendorph P, Bräuner-Osborne H (2007). Pharmacological characterization of mouse GPRC6A, an L-α-amino acid receptor with ability to sense divalent cations. Br. J. Pharmacol.

[14] Conigrave AD, Brown EM (2006). Taste receptors in the gastrointestinal tract. II. L-amino acid sensing by calcium-sensing receptors: implications for GI physiology. Am. J. Physiol.

[15] Conigrave AD, Hampson DR (2006). Broad-spectrum L-amino acid sensing by class 3 G-protein-coupled receptors. Trends Endocrinol. Metab.

[16] Conigrave AD, Mun HC, Delbridge L, Quinn SJ, Wilkinson M, Brown EM (2004). L-amino acids regulate parathyroid hormone secretion. J. Biol. Chem.

[17] Conigrave AD, Quinn SJ, Brown EM (2000). L-amino acid sensing by the extracellular Ca^2+^-sensing receptor. Proc. Natl. Acad. Sci. USA.

[18] Corbetta S, Mantovani G, Lania A, Borgato S, Vicentini L, Beretta E, Faglia G, Di Blasio AM, Spada A (2000). Calcium-sensing receptor expression and signalling in human parathyroid adenomas and primary hyperplasia. Clin. Endocrinol.

[19] Couve A, Moss SJ, Pangalos MN (2000). GABA^B^ receptors: a new paradigm in G protein signaling. Mol. Cell Neurosci.

[20] Dauban P, Ferry S, Faure H, Ruat M, Dodd RH (2000). *N^1^*-Arylsulfonyl-*N^2^*-(1-aryl)ethyl-3-phenylpropane-1,2-diamines as novel calcimimetics acting on the calcium sensing receptor. Bioorg. Med. Chem. Lett.

[21] Dupuis DS, Relkovic D, Lhuillier L, Mosbacher J, Kaupmann K (2006). Point mutations in the transmembrane region of GABAB_B2_ facilitate activation by the positive modulator *N,N*'-dicyclopentyl-2-methylsulfanyl-5-nitro-pyrimidine-4,6-diamine (GS39783) in the absence of the GABAB_B1_ subunit. Mol. Pharmacol.

[22] Fox J, Lowe SH, Conklin RL, Petty BA, Nemeth EF (1999). Calcimimetic compound NPS R-568 stimulates calcitonin secretion but selectively targets parathyroid gland Ca(2+) receptor in rats. J. Pharmacol. Exp. Ther.

[23] Fox J, Lowe SH, Petty BA, Nemeth EF (1999). NPS R-568: a type II calcimimetic compound that acts on parathyroid cell calcium receptor of rats to reduce plasma levels of parathyroid hormone and calcium. J. Pharmacol. Exp. Ther.

[24] Gama L, Breitwieser GE (1998). A carboxyl-terminal domain controls the cooperativity for extracellular Ca^2+^ activation of the human calcium sensing receptor. A study with receptor-green fluorescent protein fusions. J. Biol. Chem.

[25] Gasparini F, Kuhn R, Pin JP (2002). Allosteric modulators of group I metabotropic glutamate receptors: novel subtype-selective ligands and therapeutic perspectives. Curr. Opin. Pharmacol.

[26] Gavai AV, Vaz RJ, Mikkilineni AB, Roberge JY, Liu Y, Lawrence RM, Corte JR, Yang W, Bednarz M, Dickson JK Jr, Ma Z, Seethala R, Feyen JH (2005). Discovery of novel 1-arylmethyl pyrrolidin-2-yl ethanol amines as calcium-sensing receptor antagonists. Bioorg. Med. Chem. Lett.

[27] Gogusev J, Duchambon P, Hory B, Giovannini M, Goureau Y, Sarfati E, Drueke TB (1997). Depressed expression of calcium receptor in parathyroid gland tissue of patients with hyperparathyroidism. Kidney Int.

[28] Goudet C, Gaven F, Kniazeff J, Vol C, Liu J, Cohen-Gonsaud M, Acher F, Prezeau L, Pin JP (2004). Heptahelical domain of metabotropic glutamate receptor 5 behaves like rhodopsin-like receptors. Proc. Natl. Acad. Sci. USA.

[29] Gowen M, Stroup GB, Dodds RA, James IE, Votta BJ, Smith BR, Bhatnagar PK, Lago AM, Callahan JF, DelMar EG, Miller MA, Nemeth EF, Fox J (2000). Antagonizing the parathyroid calcium receptor stimulates parathyroid hormone secretion and bone formation in osteopenic rats. J. Clin. Invest.

[30] Hammerland LG, Garrett JE, Hung BCP, Levinthal C, Nemeth EF (1998). Allosteric activation of the Ca^2+^ receptor expressed in Xenopus laevis oocytes by NPS 467 or NPS 568. Mol. Pharmacol.

[31] Hammerland LG, Krapcho KJ, Garrett JE, Alasti N, Hung BCP, Simin RT, Levinthal C, Nemeth EF, Fuller FH (1999). Domains determing ligand specificity for Ca^2+^ receptors. Mol. Pharmacol.

[32] Hauache OM, Hu J, Ray K, Xie R, Jacobson KA, Spiegel AM (2000). Effects of a calcimimetic compound and naturally activating mutations on the human Ca^2+^ receptor and on Ca^2+^ receptor/metabotropic glutamate chimeric receptors. Endocrinology.

[33] Ho C, Conner DA, Pollak MR, Ladd DJ, Kifor O, Warren HB, Brown EM, Seidman JG, Seidman CE (1995). A mouse model of human familial hypocalciuric hypercalcemia and neonatal severe hyperparathyroidism. Nat. Genet.

[34] Hofer AM, Brown EM (2003). Extracellular calcium sensing and signalling. Nat. Cell Biol.

[35] Hu J, Jiang J, Costanzi S, Thomas C, Yang W, Feyen JH, Jacobson KA, Spiegel AM (hem.). A missense mutation in the seven-transmembrane domain of the human Ca^2+^ receptor converts a negative allosteric modulator into a positive allosteric modulator. J. Biol..

[36] Hu J, Reyes-Cruz G, Chen W, Jacobson KA, Spiegel AM (2002). Identification of acidic residues in the extracellular loops of the seven-transmembrane domain of the human Ca^2+^ receptor critical for response to Ca^2+^ and a positive allosteric modulator. J. Biol. Chem.

[37] Jensen AA, Schousboe A, Bräuner-Osborne H (2004). Molecular pharmacology of the metabotropic glutamate receptors Molecular Neuropharmacology. Strategies and Methods.

[38] Jensen AA, Greenwood JR, Bräuner-Osborne H (2002). The dance of the clams twists and turns in the family C GPCR homodimer. Trends Pharmacol. Sci.

[39] Jensen AA, Spalding TA (2004). Allosteric modulation of G-protein coupled receptors. Eur. J. Pharm. Sci.

[40] Jingami H, Nakanishi S, Morikawa K (2003). Structure of the metabotropic glutamate receptor. Curr. Opin. Neurobiol.

[41] Kessler A, Faure H, Petrel C, Rognan D, Cesario M, Ruat M, Dauban P, Dodd RH (2006). *N^1^*-Benzoyl-*N^2^*-[1-(1-naphthyl)ethyl]-*trans*-1,2-diaminocyclohexanes: Development of 4-chlorophenylcarboxamide (calhex 231) as a new calcium sensing receptor ligand demonstrating potent calcilytic activity. J. Med. Chem.

[42] Kessler A, Faure H, Petrel C, Ruat M, Dauban P, Dodd RH (2004). N2-benzyl-N1-(1-(1-naphthyl)ethyl)-3-phenylpropane-1,2-diamines and conformationally restrained indole analogues: development of calindol as a new calcimimetic acting at the calcium sensing receptor. Bioorg. Med. Chem. Lett.

[43] Kifor O, McElduff A, LeBoff MS, Moore FD Jr, Butters R, Gao P, Cantor TL, Kifor I, Brown EM (2004). Activating antibodies to the calcium-sensing receptor in two patients with autoimmune hypoparathyroidism. J. Clin. Endocrinol. Metab.

[44] Kifor O, Moore FD Jr, Delaney M, Garber J, Hendy GN, Butters R, Gao P, Cantor TL, Kifor I, Brown EM, Wysolmerski J (2003). A syndrome of hypocalciuric hypercalcemia caused by autoantibodies directed at the calcium-sensing receptor. J. Clin. Endocrinol. Metab.

[45] Kuang D, Yao Y, Lam J, Tsushima RG, Hampson DR (2005). Cloning and characterization of a family C orphan G-protein coupled receptor. J. Neurochem.

[46] Kubo Y, Miyashita T, Murata Y (1998). Structural basis for a Ca^2+^-sensing function of the metabotropic glutamate receptors. Science.

[47] Kunishima N, Shimada Y, Tsuji Y, Sato T, Yamamoto M, Kumasaka T, Nakanishi S, Jingami H, Morikawa K (2000). Structural basis of glutamate recognition by a dimeric metabotropic glutamate receptor. Nature.

[48] Miedlich SU, Gama L, Seuwen K, Wolf RM, Breitwieser GE (2004). Homology modeling of the transmembrane domain of the human calcium sensing receptor and localization of an allosteric binding site. J. Biol. Chem.

[49] Mun HC, Culverston EL, Franks AH, Collyer CA, Clifton-Bligh RJ, Conigrave AD (2005). A double mutation in the extracellular Ca^2+^-sensing receptor's venus flytrap domain that selectively disables L-amino acid sensing. J. Biol. Chem.

[50] Mun HC, Franks AH, Culverston EL, Krapcho K, Nemeth EF, Conigrave AD (2004). The Venus Fly Trap domain of the extracellular Ca^2+^-sensing receptor is required for L-amino acid sensing. J. Biol. Chem.

[51] Nelson G, Chandrashekar J, Hoon MA, Feng L, Zhao G, Ryba NJ, Zuker CS (2002). An amino-acid taste receptor. Nature.

[52] Nelson G, Hoon MA, Chandrashekar J, Zhang Y, Ryba NJ, Zuker CS (2001). Mammalian sweet taste receptors. Cell.

[53] Nemeth EF, DelMar EG, Heaton WL, Miller MA, Lambert LD, Conklin RL, Gowen M, Gleason JG, Bhatnagar PK, Fox J (2001). Calcilytic compounds: Potent and selective Ca^2+^ receptor antagonists that stimulate secretion of parathyroid hormone. J. Pharmacol. Exp. Ther.

[54] Nemeth EF, Delmar EG, Heaton WL, Miller MA, Lambert LD, Conklin RL, Gowen M, Gleason JG, Bhatnagar PK, Fox J (2001). Calcilytic compounds: potent and selective Ca^2+^ receptor antagonists that stimulate secretion of parathyroid hormone. J. Pharmacol. Exp. Ther.

[55] Nemeth EF, Heaton WH, Miller M, Fox J, Balandrin MF, Van Wagenen BC, Colloton M, Karbon W, Scherrer J, Shatzen E, Rishton G, Scully S, Qi M, Harris R, Lacey D, Martin D (2004). Pharmacodynamics of the type II calcimimetic compound cinacalcet HCl. J. Pharmacol. Exp. Ther.

[56] Nemeth EF, Steffey ME, Hammerland LG, Hung BCP, van Wagenen BC, DelMar EG, Balandrin MF (1998). Calcimimetics with potent and selective activity on the parathyroid calcium receptor. Proc. Natl. Acad. Sci. USA.

[57] Pace AJ, Gama L, Breitwieser GE (1999). Dimerization of the calcium-sensing receptor occurs within the extracellular domain and is eliminated by Cys->Ser mutations at Cys^101^ and Cys^236^. J. Biol. Chem.

[58] Peacock M, Bilezikian JP, Klassen PS, Guo MD, Turner SA, Shoback D (2005). Cinacalcet hydrochloride maintains long-term normocalcemia in patients with primary hyperparathyroidism. J. Clin. Endocrinol. Metab.

[59] Petrel C, Kessler A, Dauban P, Dodd RH, Rognan D, Ruat M (2004). Positive and negative allosteric modulators of the Ca^2+^-sensing receptor interact within overlapping but not identical binding sites in the transmembrane domain. J. Biol. Chem.

[60] Petrel C, Kessler A, Maslah F, Dauban P, Dodd RH, Rognan D, Ruat M (2003). Modeling and mutagenesis of the binding site of Calhex 231, a novel negative allosteric modulator of the extracellular Ca^2+^ sensing receptor. J. Biol. Chem.

[61] Pi M, Faber P, Ekema G, Jackson PD, Ting A, Wang N, Fontilla-Poole M, Mays RW, Brunden KR, Harrington JJ, Quarles LD (2005). Identification of a novel extracellular cation-sensing G-protein-coupled receptor. J. Biol. Chem.

[62] Pin J-P, Galvez T, Prézeau L (2003). Evolution structure and activation mechanism of family 3/C G-protein coupled receptors. Pharmacol. Ther.

[63] Prézeau L, Galvez T, Kaupmann K, Joly C, Brabet I, Froestl W, Heid J, Malischek B, Urwyler S, Pin J-P, Bettler B (1999). A single residue in GABA_-B_ receptor Type 1 is responsible for the Ca^2+^-sensing property of the GABA_-B_ receptor heteromer. Neuropharmacology.

[64] Quinn SJ, Bai M, Brown EM (2004). pH Sensing by the calcium-sensing receptor. J. Biol. Chem.

[65] Quinn SJ, Kifor O, Trivedi S, Diaz R, Vassilev P, Brown E (1998). Sodium and ionic strength sensing by the calcium receptor. J. Biol. Chem.

[66] Quinn SJ, Ye C-P, Diaz R, Kifor O, Bai M, Vassilev P, Brown E (1997). The Ca^2+^-sensing receptor a target for polyamines. Am. J. Physiol.

[67] Ray K, Hauschild BC, Steinbach PJ, Goldsmith PK, Hauache O, Spiegel AM (1999). Identification of the cysteine residues in the amino-terminal extracellular domain of the human Ca^2+^ receptor critical for dimerization. J. Biol. Chem.

[68] Ray K, Northup J (2002). Evidence for distinct cation and calcimimetic compound (NPS 568) recognition domains in the transmembrane regions of the human Ca^2+^ receptor. J. Biol. Chem.

[69] Ray K, Tisdale J, Dodd RH, Dauban P, Ruat M, Northup JK (2005). Calindol a positive allosteric modulator of the human Ca^2+^ receptor, activates an extracellular ligand-binding domain-deleted rhodopsin-like seven-transmembrane structure in the absence of Ca^2+^. J. Biol. Chem.

[70] Rey O, Young SH, Papazyan R, Shapiro MS, Rozengurt E (2006). Requirement of the TRPC1 cation channel in the generation of transient Ca^2+^ oscillations by the calcium-sensing receptor. J. Biol. Chem.

[71] Rey O, Young SH, Yuan J, Slice L, Rozengurt E (2005). Amino acid-stimulated Ca^2+^ oscillations produced by the Ca^2+^-sensing receptor are mediated by a phospholipase C/inositol 1,4,5-trisphosphate-independent pathway that requires G12, Rho, filamin-A, and the actin cytoskeleton. J. Biol. Chem.

[72] Romano C, Miller JK, Hyrc K, Dikranian S, Mennerick S, Takeuchi Y, Goldberg MP, O'Malley KL (2000). Covalent and noncovalent interactions mediate metabotropic glutamate teceptor mGlu_5_ dimerization. Mol. Pharmacol.

[73] Romano C, Yang W-L, O'Malley KL (1996). Metabotropic glutamate receptor 5 is a disulfide-linked dimer. J. Biol. Chem.

[74] Silve C, Petrel C, Leroy C, Bruel H, Mallet E, Rognan D, Ruat M (2005). Delineating a Ca^2+^ binding pocket within the venus flytrap module of the human calcium-sensing receptor. J. Biol. Chem.

[75] Slatopolsky E, Brown A, Dusso A (1999). Pathogenesis of secondary hyperparathyroidism. Kidney Int. Suppl.

[76] Tfelt-Hansen J, Brown EM (2005). The calcium-sensing receptor in normal physiology and pathophysiology: a review. Crit. Rev. Clin. Lab. Sci.

[77] Tsuchiya D, Kunishima N, Kamiya N, Jingami H, Morikawa K (2002). Structural views of the ligand-binding cores of a metabotropic glutamate receptor complexed with an antagonist and both glutamate and Gd^3+^. Proc. Natl. Acad. Sci. USA.

[78] Wellendorph P, Hansen KB, Balsgaard A, Greenwood JR, Egebjerg J, Bräuner-Osborne H (2005). Deorphanization of GPRC6A a promiscuous L-α-amino acid receptor with preference for basic amino acids. Mol. Pharmacol.

[79] Weston AH, Absi M, Ward DT, Ohanian J, Dodd RH, Dauban P, Petrel C, Ruat M, Edwards G (2005). Evidence in favor of a calcium-sensing receptor in arterial endothelial cells: studies with calindol and Calhex 231. Circ. Res.

[80] Wise A, Green A, Main MJ, Wilson R, Fraser N, Marshall F (1999). Calcium sensing properties of the GABA_B_ receptor. Neuropharmacology.

[81] Yang W, Wang Y, Roberge JY, Ma Z, Liu Y, Michael Lawrence R, Rotella DP, Seethala R, Feyen JH, Dickson JK Jr (2005). Discovery and structure-activity relationships of 2-benzylpyrrolidine-substituted aryloxypropanols as calcium-sensing receptor antagonists. Bioorg. Med. Chem. Lett.

[82] Young SH, Rozengurt E (2002). Amino acids and Ca^2+^ stimulate different patterns of Ca^2+^ oscillations through the Ca^2+^-sensing receptor. Am. J. Physiol.

[83] Zhang Z, Qiu W, Quinn SJ, Conigrave AD, Brown EM, Bai M (2002). Three adjacent serines in the extracellular domains of the CaR are required for L-amino acid-mediated potentiation of receptor function. J. Biol. Chem.

[84] Zhao GQ, Zhang Y, Hoon MA, Chandrashekar J, Erlenbach I, Ryba NJ, Zuker CS (2003). The receptors for mammalian sweet and umami taste. Cell.

